# Effect of Bifidobacterium on osteoclasts: TNF-α/NF-κB inflammatory signal pathway-mediated mechanism

**DOI:** 10.3389/fendo.2023.1109296

**Published:** 2023-03-09

**Authors:** Yue Wu, Yunjiao Yang, Lan Wang, Yiding Chen, Xuke Han, Lisha Sun, Huizhen Chen, Qiu Chen

**Affiliations:** ^1^ School of Clinical Medicine, Chengdu University of Traditional Chinese Medicine, Chengdu, China; ^2^ Department of Endocrinology, Hospital of Chengdu University of Traditional Chinese Medicine, Chengdu, China; ^3^ College of Acupuncture & Tuina, Shaanxi University of Chinese Medicine, Xianyang, China

**Keywords:** Bifidobacterium, TNF-α, NF-κB, inflammation, osteoclast

## Abstract

Osteoporosis is a systemic multifactorial bone disease characterized by low bone quality and density and bone microstructure damage, increasing bone fragility and fracture vulnerability. Increased osteoclast differentiation and activity are important factors contributing to bone loss, which is a common pathological manifestation of bone diseases such as osteoporosis. TNF-a/NF-κB is an inflammatory signaling pathway with a key regulatory role in regulating osteoclast formation, and the classical pathway RANKL/RANK/OPG assists osteoclast formation. Activation of this inflammatory pathway promotes the formation of osteoclasts and accelerates the process of osteoporosis. Recent studies and emerging evidence have consistently demonstrated the potential of probiotics to modulate bone health. Secretions of *Bifidobacterium*, a genus of probiotic bacteria in the phylum Actinobacteria, such as short-chain fatty acids, equol, and exopolysaccharides, have indicated beneficial effects on bone health. This review discusses the molecular mechanisms of the TNF-a/NF-κB inflammatory pathway in regulating osteoclast formation and describes the secretions produced by *Bifidobacterium* and their potential effects on bone health through this pathway, opening up new directions for future research.

## 1 Introduction

Physiologically and pathologically, bone volume fraction depends mainly on the rate of bone formation by osteoblasts and the rate of resorption by osteoclasts. In most pathological bone diseases such as osteoporosis, excessive bone resorption by osteoclasts is the main cause of bone loss. At present, three main categories of drugs are used for osteoporosis treatment: anti-resorptive agents (inhibits osteoclasts), bone-forming agents (boost osteoblasts), and dual-effect drugs (both promote bone formation and inhibit osteoclasts) (1). Although they have shown good clinical efficacy, their side effects cannot be ignored (2–5). For example, bisphosphonates are a type of anti-bone resorption agent that increases the survival and activity of osteoclasts mainly by inhibiting the mevalonate biosynthetic pathway or by binding to a non-hydrolyzable analogue of ATP (6). As a first-line drug for osteoporosis, bisphosphonates have demonstrated some beneficial effects, including increasing bone density and lowering the risk of fracture (7), particularly hip fracture (8). Bisphosphonates are commonly well tolerated, but side effects can occur in up to 10% of patients, mainly including arthralgia, myalgia, and gastrointestinal discomfort (9). Other negative effects including uveitis (10), atypical femoral fractures (AFFs) (11), and osteonecrosis of the jaw (ONJ) (12), are relatively uncommon. Selective estrogen receptor modulators (SERMs) and anti-RANKL monoclonal antibodies are the other two classes of anti-resorptive drugs. SERMs include raloxifene and bazedoxifene, both of which have been shown to effectively prevent bone loss and reduce bone turnover (13). However, in a three-year clinical trial, the incidence of vasodilatation (hot flashes), leg cramps and venous thromboembolic events was significantly higher in the bazedoxifene and raloxifene groups compared with the placebo group (14). The latter is represented by denosumab, a human monoclonal antibody against RANKL, a key bone resorption mediator (15). Postmenopausal women treated with the drug showed a remarkable long-term reduction in the risk of fracture of up to 10 years (16). But, after treatment was stopped, the rate of vertebral fractures rose to the equivalent in untreated people (2). As a potent bone-forming drug, teriparatide is a good candidate for improving bone microstructure (17). However, it is only used in patients with grievous osteoporosis because it requires daily subcutaneous injection and is significantly more expensive than other osteoporosis medications (18). Romosozumab is a monoclonal antibody that binds sclerostin and has a dual regulatory effect of promoting bone formation and suppressing bone resorption (19). According to the findings of the current study, romosozumab is contraindicated for individuals with a recent history of a cardiovascular incident and should only be used with caution in patients with a high cardiovascular risk (5). The safety and affordability of the drug are key concerns for patients. Therefore, how to innovate and optimize the safety and efficacy of therapeutic drugs, while keeping them affordable to osteoporosis patients is a practical clinical problem that needs to be addressed.

In recent years, probiotics have become a research hotspot. Probiotics are described as living microorganisms, which, in sufficient quantities, provide health benefits to the host (20). Probiotics have been used as preventive and curative therapy for multiple illnesses, including diabetes (21, 22), hypothyroidism (23), Hashimoto’s thyroiditis (24), and osteoporosis (25, 26). In particular, their effects on osteoporosis are even more far-reaching (25, 27, 28). Probiotic preparations are live bacterial preparations composed of probiotics or probiotic growth-promoting substances that confer health benefits to the host, using microbiological principles (29). Among them, *Bifidobacterium*, Lactobacillus, Escherichia, Enterococcus, Bacillus and Streptococcus are the most commonly bacteria used in probiotic preparations (30–32). In particular, *Bifidobacterium* inhibits osteoclast formation to ameliorate osteoporosis. Several research and clinical studies have demonstrated that, probiotics, despite their disease-prevention and treatment effects, are not absolutely safe or without side effects (33). Probiotics may be an occasional risk factor for sepsis (34). However, in general, the benefits of probiotics outweigh the disadvantages, especially the lower incidence of adverse events in *Bifidobacterium* therapy (34).

RANKL/RANK/OPG has been extensively corroborated as a classical pathway for regulating osteoclast formation, but its relationship with the TNF-α/NF-κB signaling pathway has elicited great interest in recent years. *Bifidobacterium*, a probiotic of the intestine, has profound effects on the TNF-α/NF-κB inflammatory pathway. Its secretions, including short-chain fatty acids, equol, and exopolysaccharides have distinct effects on the aforementioned inflammatory pathway. Among them, short-chain fatty acids and equol have been extensively demonstrated to exert inhibitory effects on osteoclast formation independent of this signaling pathway. This review provides an overview of the specific mechanisms of *Bifidobacterium* inhibition of the TNF-α/NF-κB inflammatory pathway to affect osteoclast formation. Through this review, we attempt to provide researchers with new insights into potential targets for the development of effective therapies for osteoporosis.

## 2 TNF-a/NF-κB signaling pathway

TNF-α/NF-κB is a well-known inflammatory signaling pathway (35) that is implicated in the development of endocrine system illnesses, particularly osteoporosis (36, 37). The tumor necrosis factor (TNF) superfamily molecules are mostly produced by macrophages (38). Among them, TNF-α is a vigorous pro-inflammatory cytokine with a crucial role in immune function, inflammation, and regulation of cell growth, differentiation, and apoptosis (39). TNF-α requires cell surface receptors tumor necrosis factor receptor 1 (TNFR1) and tumor necrosis factor receptor 2 (TNFR2) to exert its biological effects (40). Whereas TNFR1 and TNFR2 have extracellular domains enriched with cysteine, their intracellular domains are structurally very different. Notably, TNFR1 contains a conserved 80-amino acid sequence called the cytoplasmic “death domain,” which produces a characteristic fold (41). Through this death structural domain, TNFR1 can sequentially recruit tumor necrosis factor receptor-associated death domain protein (TRADD), TNFR-associated factor 2 (TRAF2), receptor-interacting protein, and nuclear factor-κB (NF-κB) kinase inhibitor (IKK), thereby activating NF-κB (40). Contrarily, TNFR2 does not have a cytoplasmic death region sequence and recruits TNFR-associated factor 1 (TRAF1) and TNFR-associated factor 2 (TRAF2), but not TRADD (42). Despite this difference, the signaling cascades downstream of TNFR1-TRADD-TRAF2 and TNFR2-TRAF2 are similar.

**Figure 1:**
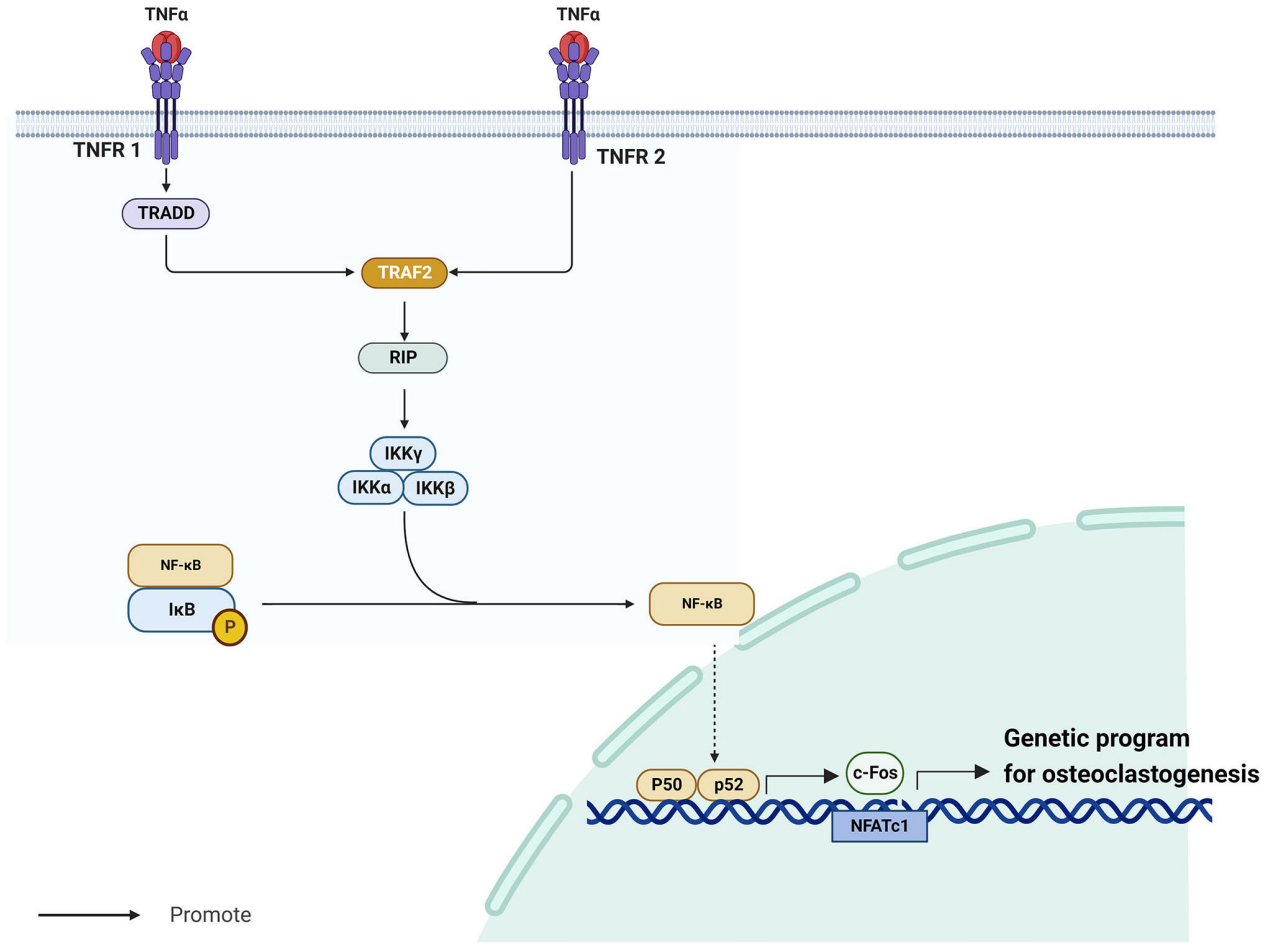
TNF-α/NF-κB signaling pathway.

NF-κB is a homodimeric and heterodimeric complex composed of five members of the Rel family, including NF-kB1 (p50), NF-kB2 (p52), RelA (p65), RelB, and c-Rel (43). These factors regulate the expression of several genes involved in immune response and numerous other cellular processes, including growth, development, and apoptosis (44). In most unstimulated cells, IkB proteins are maintained inactive in the cytoplasm by interacting with NF-kB dimers (45). Kappa B inhibitor kinase (IKK) is a heterotrimeric enzyme made up of the kinase subunits IKKa and IKKb as well as the regulatory subunit IKKγ/NEMO (46). When activated, IKK phosphorylates and degrades two important serine residues in the N-terminal regulatory domain of the NF-κB inhibitor IkB, releasing NF-κB (47). After its release, NF-κB generates cytokines such as p50 and p65, which promote its translocation to the nucleus to revive transcription (47). When NF-κB p50 and p52 are expressed, RANKL-RANK induces osteoclastogenesis (36). NF-κB promotes the activation of c-Fos (48), a member of the Fos gene family, which together with Jun proteins make up the AP-1 family of heterodimeric transcription factors (49). Without c-Fos, osteoclasts cannot develop (50). Boyce et al. demonstrated that c-Fos primarily generates and interacts with NFATc1 to initiate a transcriptional regulatory cascade, which results in upregulation of several target genes involved in osteoclast development and function (51). The TNF-α/NF-κB signaling pathway is in **Figure 1**.

## 3 RANKL/RANK/OPG signaling pathway

RANKL/RANK/OPG is the predominant signaling pathway regulating osteoclast differentiation (52). The differentiation of osteoclasts primarily involves the fusion of monocytes to multinucleated osteoclasts in response to three cytokines: macrophage colony-stimulating factor (M-CSF), nuclear factor-κB (NF-κB) ligand receptor activator (RANKL) and osteoprotegerin (OPG) (25). M-CSF (also known as colony-stimulating factor-1) regulates mononuclear phagocyte production, through a process mediated by CSF-1 receptor (CSF-1R), which is encoded by the c-FMS proto-oncogene (53). As a dimeric cytokine, it modulates the formation of many different types of cells, such as trophoblasts, macrophages, and osteoclasts (54). In the early stage of osteoclast development, M-CSF binds to c-FMS expressed on precursor cells activating their proliferation (53). A type II membrane protein with close homology to the TNFSF members TRAIL, FasL, and TNF-a (55), RANKL is expressed on osteoblasts as a membrane-associated cytokine (56). *In vitro*, RANKL activates mature osteoclasts in a dose-dependent manner, but can activate pre-existing osteoclasts quickly to cause bone resorption *in vivo* (57, 58). Additionally, it has been demonstrated that M-CSF and RANKL promote the differentiation of osteoclast precursor cells into mature and functional osteoclasts (56, 59). Collectively, CSF-1 and RANKL can stimulate the expression of genes that characterize the osteoclast lineage, including those that encode tartrate-resistant acid phosphatase, cathepsin K, and calcitonin receptor, resulting in the maturation of osteoclasts (60). Through intercellular contacts, osteoblasts express RANKL, which is recognized and binds to osteoclast precursors, which then develop into osteoclasts in the presence of M-CSF (61). Meanwhile, M-CSF strongly promotes the binding of RANK to RANKL and the formation of osteoclasts (62).

Osteoprotegerin (OPG) is a cytokine receptor protein produced by osteoblasts (63). It acts as a decoy receptor by binding to RANKL to block its interaction with its functional receptor RANK, thereby inhibiting osteoclast formation (64). OPG has also been found to cause osteoclast pseudopod disassembly and safeguard the bone cortex *via* pathways like the Ca-p38-MAPK signaling pathway, inhibit RANKL binding to RANK, prevent osteoblast-induced osteoclast precursor cell differentiation, and control osteoclast function (65). Li et al. demonstrated that cytokines (such as OPG and RANKL) directly interact with bone regulating proteins to enhance bone homeostasis (66). Another study confirmed that dexamethasone-induced osteoporosis can be improved by restoring OPG expression by decreasing RANKL expression (67). Hence, the ratio of OPG/RANKL determines the degree of bone resorption and the course of bone metabolism.

## 4 TNF-α/NF-κB signaling pathway and RANKL/RANK/OPG signaling pathway

Inflammation is closely linked to osteoporosis. TNF-α is a potent pro-inflammatory cytokine (39), and IL-6 is a “classical” bone resorption pro-inflammatory cytokine (68). It has been demonstrated that IL-1, a pro-inflammatory cytokine, promotes osteoclast production, which in turn stimulates bone resorption (69). TNF-α stimulates inflammatory cytokine mRNA transcription, which results in the production of IL-6 (70). It has been discovered that recombinant human tumor necrosis factor (rTNF-α) naturally induces IL-1 in the body (71). Furthermore, IL-1 can induce the expression of TNF-α *via* an autocrine mechanism (72) and IL-1 induces the production of IL-6 (73). Thus, inflammatory cytokines can not only promote bone resorption alone, but their mutual activation can enhance the activation of TNF-α/NF-κB signaling pathway, activate osteoclast-related genes, and enhance bone resorption, which can be seriously detrimental to osteoporotic patients. The cytokines TNF-α, IL-6, and IL-1 cause a significant augmentation of osteoclasts and a suppression of osteoblast activity when RANKL is present (74). IL-17 is an another pro-inflammatory cytokine that promotes bone resorption *via* upregulating RANKL (75). IL-6 trans-signaling directly increases RANKL on fibroblast-like synovial cells and is involved in the induction of RANKL by TNF and IL-17 (76). In addition, IL-6 and TNF-α can synergistically activate NF-κB (77). Ciucci et al. further ascertained that bone marrow CD4^+^ T cells belong to a distinct subpopulation of osteoclastic T cells termed Th17 TNF-α (+) cells that can generate IL-17 and TNF-α (78). These cells move to the bone marrow amid chronic inflammation, where they facilitate the recruitment of inflammatory monocytes (mainly osteoclast progenitors) (78). In an inflammatory event, immune system cells, such as T cells, B cells, macrophages, and dendritic cells, become activated and release inflammatory cytokines, which are among the most crucial mediators in bone immunology (74). Activated T cells are particularly significant mediators because they increase the production of the so-called bone resorbing cytokines, including TNF- and RANKL (74). Thus, the formation of osteoclasts is closely associated with chronic inflammation. In addition to stimulating osteoclast formation through the NF-κB signaling pathway, TNF-α can also mediate RANK ligands activation of osteoclast formation *via* an autocrine mechanism (79). The combination of TNF-α and RANKL greatly stimulated osteoclast formation and significantly up-regulated osteoclast mRNA markers (37).

**Figure 2:**
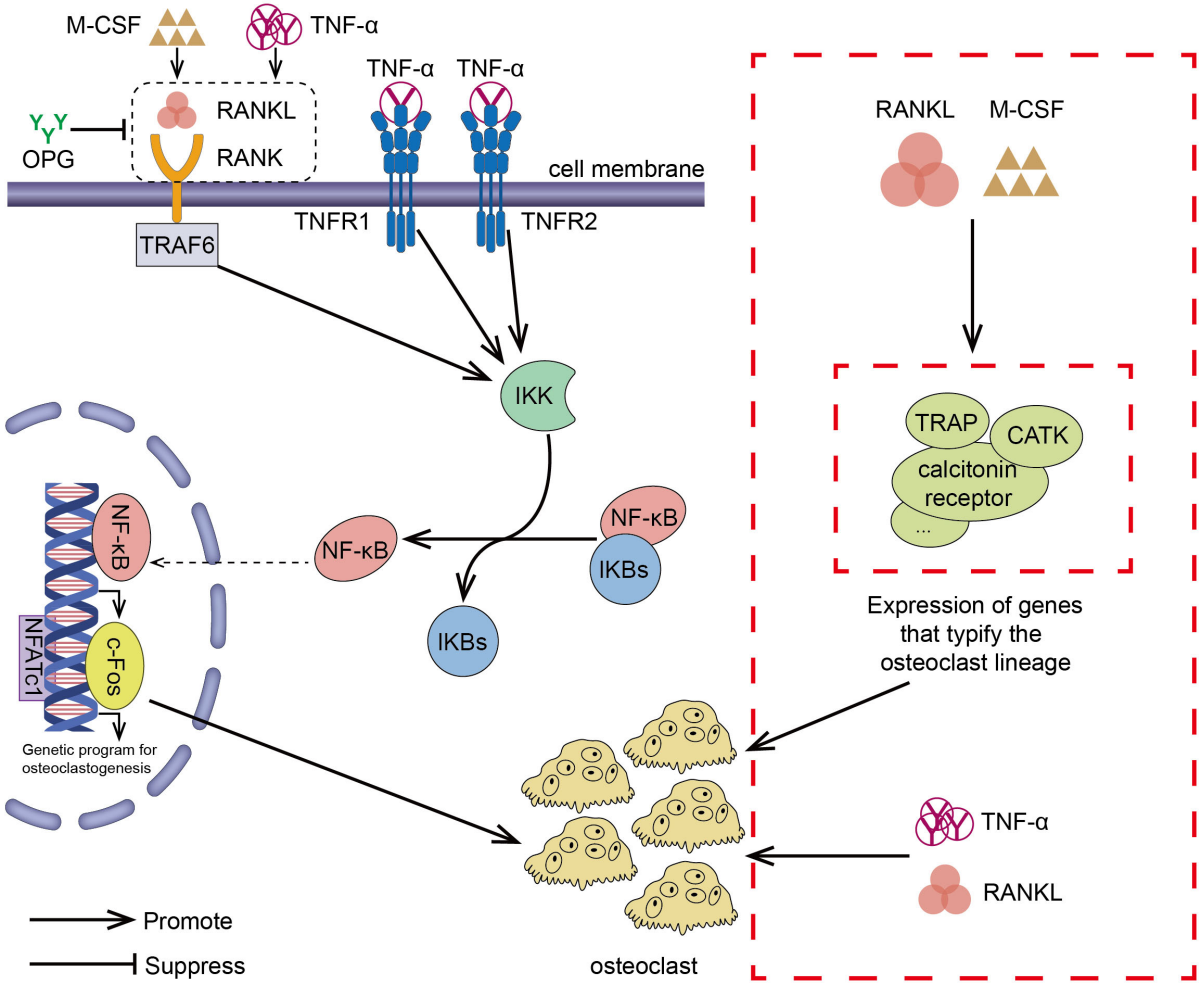
RANKL/RANK/OPG is the most important signaling pathway regulating osteoclasts formation. The interaction between RANK and RANKL (which can exacerbate this effect in the presence of M-CSF) promotes the recruitment of the TRAF family bridging proteins, one of which TRAF6 contributes to OC formation and activation of the NF-κB signaling pathway, leading to the transcription of genes involved in OC formation and OC production. OPG is a decoy receptor that binds RANKL and can block the binding and activation of RANK and RANKL, reducing OC production. TNF-α/NF-κB is an inflammatory signaling pathway. In the presence of TNF-α, NF-κB pathway is activated, OC production is increased, and the interaction between RANKL and RANK is enhanced, which results in activation of the downstream signaling pathways. In addition to the above-mentioned effects, TNF-α also synergizes with RANKL and directly promotes OC production. In the presence of RANKL and M-CSF, the expression of genes involved in OC formation leading to the development of mature OC.

To sum up, inhibiting the TNF-α/NF-κB inflammatory pathway can impede the formation of osteoclasts. Multiple studies (80, 81) have demonstrated a strong link between inflammation and osteoclast formation. The important role of the NK-kB transcription factor family in inflammation and innate immunity has also been elucidated (82, 83). Inhibition of osteoclast formation *via* the NF-kB pathway has also been reported. For example, preparations of *Zanthoxylum piperitum* (84), *Sophorae flos (* 85), and *Bajijiasu (* 86), were shown to inhibit the RANKL-induced NF-κB/NFATc1 pathway in osteoblasts to hinder bone resorption. As a pro-inflammatory cytokine, TNF-α promotes the production of osteoclasts by activating the NF-κB pathway, synergizing RANKL cytokines, and facilitating and enhancing RANK-RANKL binding. In periodontitis, down-regulating TNF-α, alveolar bone loss was delayed (87). In addition, Yao et al. explained the regulation of TNF-α-induced osteoclast formation (88). The effect of RANKL/RANK/OPG on osteoclasts has also been confirmed by many investigators (52, 89, 90). TNF-α/NF-κB signaling pathway and RANKL/RANK/OPG signaling pathway induced-osteoclast have been shown in **Figure 2**.

In recent years, the impact of probiotics on bone health has become a hotspot of research. Current research suggests that probiotics regulate bone metabolism through different mechanisms, including intestinal barrier permeability (91), metabolite production (92), the immune response (93), and inflammation (94). Postmenopausal bone mineral density loss, which is associated with estrogen deficiency (95), was effectively reduced by probiotic supplementation, which also improved bone turnover in 78 postmenopausal patients with osteoporosis for more than a year (96). In another trial, probiotics significantly increased total hip bone density compared with placebo and modulated gut microbiota in postmenopausal women (97). In response to the aforementioned pathways, recent reports show that probiotics, particularly *Bifidobacterium*, have more benefits for bone health (**Table 1**).

**Table 1:** Beneficial effects of related Bifidobacterium strains on bone health.

Related Bifidobacterium strains	Sex	Experiment model	Duration of intervention	Bone effects	Reference
Bifidobacterium longum NK49	Female	C57BL/6 mice	2 weeks	↑Serum levels of Ca and P ↑Serum levels of Osteocalcin ↑IL-10 ↓TNF-α ↓NF-κB	(98)
Bifidobacterium longum ATCC 15707	Male	Wistar rats	28 days	↑Tibial Ca, P, and Mg content ↑Fracture strength ↑SCFAs concentration	(99)
Bifidobacterium animalis subsp. Lactis	Male	Wistar rats	15 days	↓TRAP-positive multinucleated cells ↓The number of osteoclastes ↓IL-1β ↑IL-10	(100)
Bifidobacterium longum ATCC 15707	Female	Sprague-Dawley rats	16 weeks	↑BV/TV ↑Tb.N ↑Tb.Th ↑BMD ↑Serum levels of Osteocalcin ↓Serum levels of C-terminal telopeptide	(101)
Bifidobacterium longum–fermented broccoli	Male	Wistar rats	12 weeks	↓TRAP-positive osteoclasts	(102)
Bifidobacterium animalis Subsp Lactis	Male	Wistar rats	2 weeks	↑BV ↑OPG ↑IL-10 ↓Bone loss ↓RANKL ↓IL-1β ↓RANKL/OPG ratio ↓IL-1β/IL10 ratio	(103)
Bifidobacterium longum UBBL-64 (M1395)	Female	C57BL/6 J mice	6 weeks	↑IL-10 ↓The number of multinucleated (>3 nuclei) TRAP-positive cells ↓The number and area of F-actin rings ↓TNF-α ↓IL-6 ↓IL-17	(93)
Bifidobacterium adolescentis	Male	C57BL/6 J mice	36days	↑Fracture healing ↑Tight junction genes expression ↓Inflammation	(104)

↑, means to increase; ↓, means to reduce.

## 5 Bifidobacterium

**Figure 3:**
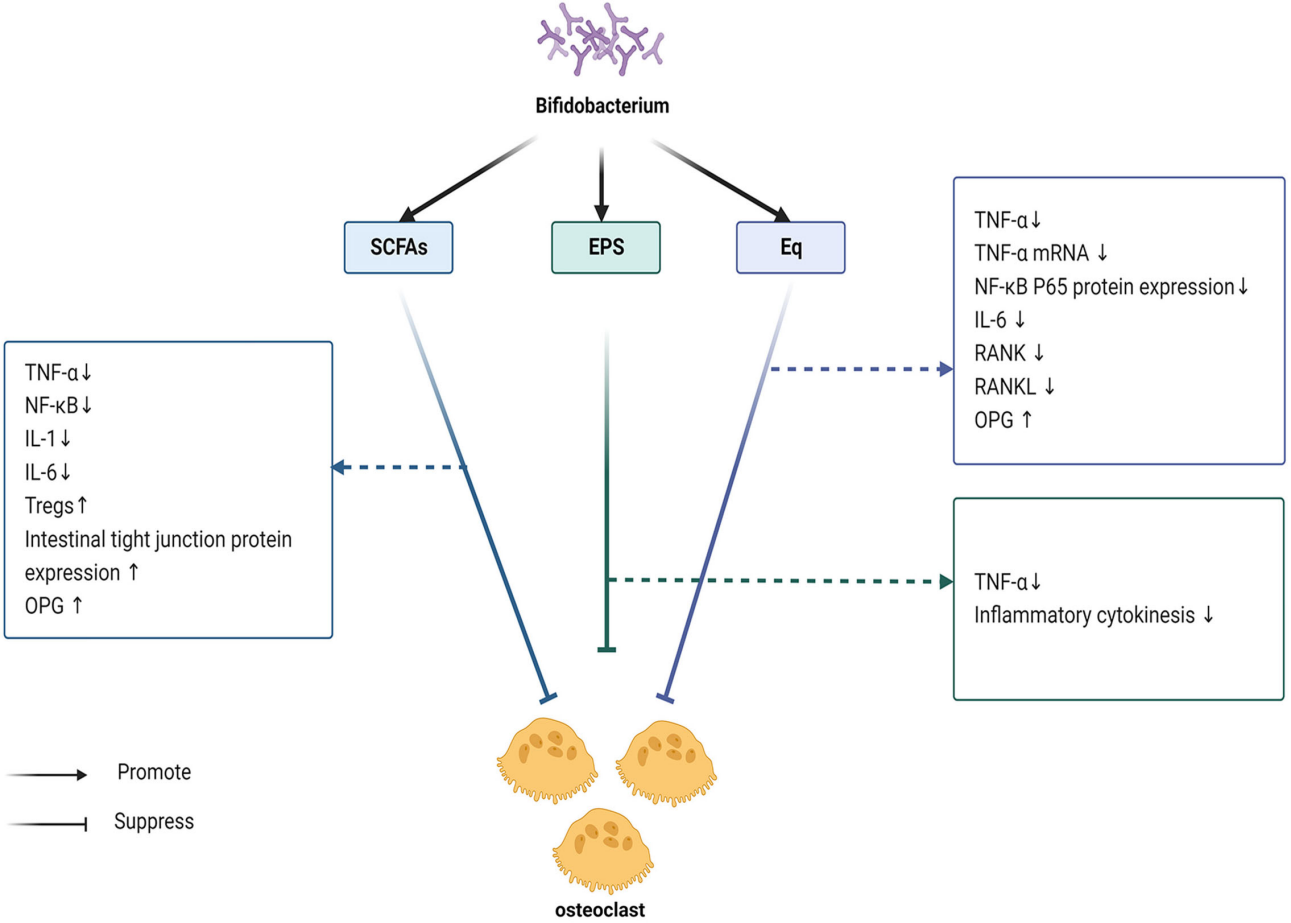
Bifidobacterium mainly produces SCFA, Eq and EPS. These three substances inhibit the TNF-α/NF-κB signaling pathway, reduces the production of inflammatory mediators, and blocks the activation of inflammatory mediators, thereby preventing the formation of OC. SCFA can promote the production of Treg cells, indirectly inhibit the TNF-α/NF-κB signaling pathway, and also regulate OC formation through Treg cells. In addition, SCFA plays a role in the maintenance of the intestinal mucosal barrier, blocking the entry of inflammatory factors into the bloodstream, and reducing inflammation, leading to the inhibition of OC formation. More importantly, it has been shown that SCFA also increases the production of OPG. Eq also decreases the production of inflammatory factors, such as IL-6, inhibits RANK and RANKL, and can upregulate OPG expression. EPS mainly inhibits the production of inflammatory factors.

The most prevalent phyla of the human gut microbiota are Firmicutes, Bacteroidetes, and Actinobacteria (105). The phyla Firmicutes and Bacteroidetes collectively account for 90% of colonic microbiota (60–75% and 30–40%, respectively) (106, 107). By comparison, the phylum Actinomycetes is a smaller but essential component for preserving intestinal homeostasis (108). *Bifidobacterium*, which belongs to the phylum Actinobacteria (105), was first isolated from the feces of healthy breastfed infants by a French pediatrician Tissier and named *Bifidobacterium* because of its commonly bifurcated ends (109). *Bifidobacterium* can produce short-chain fatty acids (SCFA) (110), equol (Eq) (111), exopolysaccharides (112) and many other substances that can affect osteoclast formation, which is shown in **Figure 3**.

### 5.1 Short chain fatty acids

SCFAs are molecules less than 6 carbon atoms in size (C1-C6) (113). Intestinal bacteria degrade dietary fiber into products such as butyrate, propionate, and acetate so on, which are collectively called SCFAs (114). Acetate, fructose, and lactate can be produced during the fermentation of dietary fiber by *Bifidobacterium*, but not butyrate (115). However, in hybrid cultures composed of *Bifidobacterium* and *E. coli* or *A. caccae*, the addition of dietary fiber results in the production of butyrate (115). This study shows that SCFAs not only directly affect the TNF-α/NF-κB inflammatory pathway, but also inhibit TNF-α/NF-κB-mediated inflammation by promoting the secretion of regulatory T cells and increasing intestinal tight junction protein expression, thereby controlling osteoclast formation. In addition, osteoclasts can be regulated by Treg cells independent of this pathway. Of course, the direct effect of **SCFAs** on osteoclasts cannot be ignored.

There are substantial reports on anti-inflammatory properties of SCFAs, including the results of basic experiments done to inhibit the TNF-α/NF-κB inflammatory pathway. At low concentrations, acetic acid, propionic acid, and butyric acid exert potent anti-inflammatory effects by inhibiting the generation of pro-inflammatory agents, such as NO, TNF-α, IL-1, and IL-6 (116). Downar et al. showed that pretreatment of human umbilical vein endothelial cells with butyric acid suppressed TNF-α-induced activation of NF-κB (117). In another experiment, all SCFA**s** dose-dependently decreased NF-κB reporter activity in Colo320DM cells and 30 mmol/L of acetate, propionate, and butyrate reduced LPS-stimulated TNF-α release from neutrophils (118). Certainly, by directly blocking NF-kB, SCFA**s** may also alleviate *S. aureus*-induced inflammatory response (119). In mice maintained in germ-free environment and fed with 150 mM SCFA *via* drinking water for three weeks, SCFA, either alone or in combination (SCFA mixture), was found to increase the frequency and amount of cTreg (120). Foxp3+ CD4+ regulatory T cells (Tregs) are a subset of immune cells that regulate tissue inflammation (121). Treg cells can restrain the function of effector T cells, thus reducing the output of TNF-α (122). They also produce interleukin-10 (IL-10), which limits environmental interface inflammation (123) and inhibits osteoclastogenesis (124). Bogdan et al. demonstrated that recombinant mouse IL-10 effectively inhibited the capacity of murine peritoneal macrophages to release TNF-α (125). The anti-inflammatory properties of Treg cells not only confer an advantage, but they can also affect osteoclast formation independent of the TNF-α/NF-κB pathway. Tregs cells can also inhibit macrophage colony-stimulating factor and RANKL to promote osteoclast formation in a dose-dependent manner (126). Compared with wild-type litter controls, FoxP3-Tg mice had higher bone mass, indicating that Treg cells can regulate bone resorption *in vivo* (127). However, no reduction in osteoclast production was seen when butyric acid or propionic acid was added 24 or 48 hours after osteoclast differentiation, and acetate alone had no significant effect on osteoclast differentiation (128). Thus, SCFAs may inhibitory effect on osteoclast production but not on osteoclasts that have already been generated.

TNF-α, which may be linked to pathogenic intestinal inflammation, can affect the shape and function of tight junctions, impairing epithelial barrier function (129). Damage to the intestinal mucosal barrier aggravates the TNF-α/NF-κB-induced pro-osteoclastogenic pathway, creating a vicious cycle of inflammation and epithelial injury. Therefore, maintenance of good integrity and proper permeability of the intestinal mucosal barrier is crucial to intestinal health and protection from other diseases. In particular, intestinal permeability is maintained in normally functioning tight junctions (130). The expression of tight junction proteins, which are essential for preserving intestinal epithelial permeability, is elevated in the presence of SCFAs (131, 132). Damage to the tight junction barrier allows toxic materials to enter the body, which can cause inflammation and over-activation of the mucosal immune system (133). Additional evidence indicates that SCFAs play an important protective role in the intestinal mucosal barrier. Intestinal development in piglets may be aided by gastric infusion of SCFAs, particularly at high SCFAs concentrations, by improving intestinal shape, lowering the percentage of apoptotic cells, and maintaining intestinal barrier function (134). Another study demonstrated that oral or direct enteral drip treatment with SCFAs enhanced the proliferation of intestinal epithelial cells (135). As a result, SCFAs play a key role in maintaining intestinal epithelial stability, reducing inflammation, and preventing osteoclast formation.

SCFAs have also been shown to directly regulate osteoclasts. By stimulating human osteoblasts to produce more OPG, sodium butyrate inhibited the development of osteoclasts (136). Nonetheless, treatment with butyrate increased cell cycle arrest and drastically diminished cell proliferation in MG-63 osteoblasts (137). Therefore, the effect of SCFAs on the formation of osteoblasts are still inconclusive. However, its inhibitory effect on bone resorption in cellular experiments or animal experiments is well supported. In RAW264 cells, sodium butyrate blocks the expression of osteoclast-specific mRNA and nuclear factor-κB (NF-κB) ligand (RANKL) receptor activator-stimulated osteoclast formation (138). Mice treated with SCFAs and fed a high-fiber diet had much more bone mass and were protected from inflammation-induced bone loss (139).

In summary, SCFAs play an important role in inhibiting the TNF-α/NF-κB inflammatory signaling pathway, regulates osteoclast formation in other ways, and exerts a considerable direct effect on osteoclasts.

### 5.2 Equol and TNF-α/NF-κB signaling pathway

Equol, with a chemical formula C15H14O3, was initially discovered and clarified by Marrian and Haslewood (140). Equol, a soy glycosides metabolite, is categorized as a polyphenolic compound (isoflavone found in plants and foods) (141). In comparison to soy sapogenins, equol has greater estrogenic activity and a stronger affinity for estrogen receptors (142). Soybean isoflavones have been shown to improve ovx-induced osteoporosis (143). They can generate the metabolite equol in the intestine, which exerts therapeutic effect on bone metabolism (144).

Equol had been demonstrated to suppress the activation of the TNF-α/NF-κB inflammatory pathway by several authors. In addition, like SCFAs, it has been shown to exert anti-inflammatory effects independent of this pathway, further supporting its role in the inhibition of osteoclast formation. Equol dramatically reduces the level of pro-inflammatory cytokine TNF-α in mice treated with lipopolysaccharide (LPS) (145, 146). Additionally, in LPS-stimulated murine macrophages, equol dose-dependently reduced TNF-α production and TNF-α mRNA expression (146). Moreover, equol may drastically lower NF-κB P65 protein expression by suppressing the activation of the NF-κB pathway (147). Subedi et al. showed that treatment with equol decreased LPS-induced production of pro-inflammatory cytokines (such as TNF-α and IL-6) and treatment of cells (pretreated with LPS) with equol at doses of 10 and 20 µM, significantly suppressed NF-κB activity (148). Among them, IL-6 is a classical factor that promotes osteoclast formation (68), which further supports the inhibitory effect of equol on osteoclast formation.

Meanwhile, its direct regulation of osteoclasts has also received much scholarly attention in recent years. Equol (0.5 mg/day subcutaneously) treatment prevented bone loss in the femur and other bones in body of ovx mice (149). In another study, however, it was discovered that equol had no particular benefit on whole-body bone density, but had a special advantage in the femur, where it inhibited bone loss throughout, proximally and distally (150). The substance probably works better on some parts of the bone than others. But it is certain that equol plays a key role in promoting bone healing and inhibiting osteoclast formation. In severe osteoporosis presenting 10 weeks after oophorectomy, the administration of equol intervention promoted fracture healing by enhancing bone trabecular structure and raising endosteal healing tissue (151). More importantly, it reduced the expression of specific genes (e.g. *Fos*) in osteoblasts (152). In the classical pathway of osteoclast formation, qRT-PCR confirmed that treatment of ovx-induced rats with equol revealed decreased RANKL and RANK mRNA expression levels and upregulated OPG expression levels (153). Thus, Eq can regulate the balance between OPG and RANKL and inhibit bone loss caused by osteoclasts.

### 5.3 Exopolysaccharides and TNF-α/NF-κB signaling pathway

Exopolysaccharides (EPS) are lengthy polysaccharide chains that are loosely linked to the microbial cell wall and thus can easily be discharged into the nearby local milieu (154). The effect of exopolysaccharides on this signaling pathway is still relatively limited, but it has been found that it can exert an inhibitory effect on osteoclasts to some extent.

The inhibition of inflammation by EPS is bidirectional. Large molecular weight and neutrally charged EPS exert their immunosuppressive effects by preventing the release of pro-inflammatory molecules (112). EPS with small molecular weights and negative charges can boost the immune system by prompting immune cells to release cytokines, including IL-10, IL-12, and TNF-α (155). *Bifidobacterium 35624*, which produces EPS, more effectively suppresses the pro-inflammatory response compared with *Bifidobacterium 35624*, which is deficient in EPS (156). And, this study showed that the former can reverse the increase in the pro-osteoclastogenic cytokine IL-17, which is induced in the absence of EPS (156). One recent study suggests that EPS acts mainly by preventing the fusion of early osteoclast precursors, without significantly affecting the resorptive activity of mature osteoclasts (154). In contrast, mice that received peroxisulfated exopolysaccharides (OS-EPS) had more osteoclasts on the surface of their trabecular bones (157). Notably, *in vitro*, the early stage of osteoclast precursor adhesion was prevented by OS-EPS, thereby preventing the cell fusion stage (157). Therefore, the effects of extracellular polysaccharides on osteoclasts are still inconclusive, and further research is needed to explain these relationships and explore the conditions under which extracellular polysaccharides are beneficial for bone health.

## 6 Analysis and future outlooks

Osteoporosis has become a major global public health problem, with a significant economic burden on health care systems. It is a bone disease that is characterized by low bone mass and microstructural degradation, which promote bone fragility and, consequently, increase the fracture risk (158). In previous studies (25, 159), the therapeutic potential of probiotics in bone health has been demonstrated, including their positive effect on osteoporosis. However, few studies have examined the relationship between specific genera of bacteria and osteoporosis, and certainly even fewer articles have discussed their specific mechanisms of action. Therefore, in this paper, we sought to fill this gap by reviewing the mechanisms of *Bifidobacterium* inhibition of the TNF-α/NF-κB inflammatory pathway to prevent osteoclast formation. In germ-free mice, higher bone mass is linked to changes in the immunological state, which is reflected by decreased expression of inflammatory cytokines in bone (25). *Bifidobacterium* can be used to inhibit the formation of osteoclasts by altering the inflammatory immune status of bones through its secretory products.

SCFAs can directly inhibit the classical pathway of inflammation, the TNF-α/NF-κB signaling pathway, to strongly inhibit inflammation. It can also promote the secretion of Tregs cells, regulate inflammation through the immune system, increase the expression of intestinal tight junction proteins, block the invasion of harmful substances through the intestinal mucosa, and effectively control inflammatory response. In the immune system, regulatory T cells (Treg cells) expressing the transcription factor Foxp3 have been shown to act as inhibitors of inflammatory response in the gut, and helper T cells 17 (Th17) are pro-inflammatory cells (160). Under inflammatory conditions, Foxp3 expression on Treg cells is lost, and this allows transdifferentiate of the cells into Th17 cells (161). The balance between Th17 cells and Treg cells influences the pathogenesis of osteoporosis. Increased Th17 cell frequency has been linked to the occurrence of bone resorption (162). Th17 also secretes high quantities of IL-17, NF-kB ligand receptor activator (RANKL), and TNF as well as low levels of interferon gamma, making it the most osteolytic subpopulation of T CD4^+^ cells (IFNγ) (163, 164). A subpopulation of immune cells known as Treg cells inhibits the differentiation and functionality of Th17 cells (165). In addition, Treg cells can inhibit OC differentiation and bone resorption by releasing TGF-β1 and IL-10 (166). It has been reported that enrichment of SCFA-producing probiotics downregulates intestinal epithelial permeability and restores the Treg/Th17 cell ratio (27). Butyric acid, in particular, is implicated in the control of Treg/Th17 balance and prevents the formation of inflammation in colonic mucosa (167). This protective effect on the intestinal mucosa contributes to the reduction in inflammation and formation of osteoclasts. These effects are mediated by the TNF-α/NF-κB inflammatory pathway. For instance, Th17 secrete high levels of NF-kB ligand receptor activator (RANKL) and TNF, and Treg inhibits Th17 cells thereby indirectly regulating the TNF-α/NF-κB signaling inflammatory pathway. Treg also inhibits osteoclast formation, and further studies are needed to expand our understanding on this. High concentration of SCFAs can inhibit bone growth. By blocking osteoblast-specific factors, high dose of sodium butyrate prevents the differentiation and mineralization of the ROS17/2.8 rat osteoblast line (168). Therefore, further investigations are advocated to determine the optimal concentration of SCFAs for the treatment of osteoporosis. On the side, butyrate also has the ability to modulate bone anabolic metabolism through Treg cell-mediated generation of Wnt10b from CD8+ T cells (169). This demonstrates the ability of short-chain fatty acids in bone formation, and perhaps *Bifidobacterium* could be drugs that have the dual effect of stimulating bone formation and inhibiting bone resorption.

There have been several reports about the effect of SCFAs on osteoclast formation (128, 136, 139). However, few scholars have explored the effect of equol and exopolysaccharides on osteoclast formation. Currently, few microorganisms have been identified to produce equol, and *Bifidobacterium* is one of them (111). Equol can inhibits the TNF-α/NF-κB pathway to exert anti-inflammatory effects and reduce the pro-osteoclastogenic function of inflammatory cytokines (145–148, 170). Ovariectomy model (OVX) mice showed significantly reduced bone mineral density (BMD) and bone mineral content (BMC) compared with sham-operated animals, and 0.5 mg/d Eq treatment preserved bone mass (149). Furthermore, it has stronger estrogenic activity and may be a potential agent for treatment of postmenopausal osteoporosis (171). Equol is produced in the gastrointestinal system by soy glycosides, however, its metabolism in humans differs among individuals (172). This may alter the efficacy of the drug leading to different responses in various patients with osteoporosis, but this concept has not been sufficiently studied. Therefore, further clinical evaluation and analysis is still needed. *In vitro* experiments have demonstrated that EPS can potentially prevent osteoclast formation, however, the optimal way to use EPS in humans that guarantees stable inhibition of osteoclast formation needs to be further explored (154).

Currently, *Bifidobacterium* is now an important product in the market. Compared with other microbial workhorses, engineered *Bifidobacterium*’s produces numerous bioproducts with additional benefit while using fewer resources (173). The Bifid shunt, which generates higher number of CoA predecessors for the bioproduction of polyketide products and fatty acid biosynthesis, is one of the crucial metabolic processes in *Bifidobacterium* (174). Although clostridia can also produce SCFAs and Eq (111, 175), it is thought to cause pathogenicity (176). Safety assessment of *Bifidobacterium* species identified only 2 cases of mild functional intestinal obstruction (177) and 1 case of sepsis (178), and these results demonstrate that probiotic preparations possess pathogenic risks. Therefore, there is need to balance between the risk and the cost-benefit and safety in the clinical treatment of patients to reduce the incidence of adverse events.

Inflammatory bowel disease (IBD) has been linked to increased risk of bone mineral loss and osteoporosis (179). *Bifidobacterium lactis BL-99* can be used to prevent the development of osteoporosis in patients with ulcerative colitis (UC) by shaping the intestinal flora and inhibiting the production of inflammatory cytokines (180). Probiotics have shown potential for the treatment of IBD, and therefore, the authors suggest that fecal transplants could be used in the future to regulate intestinal flora to improve the symptoms of IBD (181).. Studies have demonstrated that extensive changes occur in the structure of the intestinal flora of rats after ovariectomy (182). Compared with healthy individuals, patients with osteoporosis or bone loss showed significant changes in gut microbial species (183). This suggested a correlation between osteoporosis and the constitution as well as functionality of the intestinal flora. It is possible that fecal colony transplantation technique may be an effective treatment for patients with osteoporosis. However, FMT is processed by collecting therapeutic stools from normal individuals and its treatment success depends largely on the quality of the donor’s gut microbes. Therefore, appropriate selection of donors is crucial. Nevertheless, there are many challenges affecting the adoption of this technology (184). Further animal experiments and clinical studies are needed to clarify this.

## 7 Conclusion

In this review, we summarized the mechanisms by which *Bifidobacterium* bifidum regulates osteoclast formation by inhibiting the TNF-α/NF-κB inflammatory pathway. Its effects are mediated by its secreted products, including short-chain fatty acids, equol, and exopolysaccharides.

The role of probiotics in osteoporosis is increasingly being studied. Activation of inflammatory factors associated with osteoporosis such as TNF-α, NF-κB, IL-1, IL-6, and IL-17 has been to be involved in the physiology of pro-osteoclast formation. Inhibition of the TNF-α/NF-κB signaling pathway prevented nuclear transfer of NF-κB and blocked the transcription of regulatory proteins associated with osteoclasts. *Bifidobacterium* secretions block the initiation of inflammation and inhibits osteoclast formation to improve osteoporosis symptoms. In addition, the *Bifidobacterium* secretions can regulate RANKL/RANK/OPG, the most important signaling pathway of osteoclasts, *via* the TNF-α/NF-κB signaling pathway, which block the synergistic effect of TNF-α on RANKL and reduce the binding of RANK to RANKL to regulate the formation of osteoclasts. In this way, it prevents bone loss caused by the bone resorption induced by osteoclasts. Although clinically effective osteoporosis treatment drugs are available, their safety and efficacy are not satisfactory, especially in patients with severe osteoporosis. Therefore, there is a need to actively search for more effective treatments with fewer side effects and more cost-effective. The data described in this review demonstrated that *Bifidobacterium* might be a good treatment agent.

## Author contributions

YW and YY conceived the study and finished drafting the article. LW and YC polished up the article. XH, LS, and HC processed the table and pictures. QC provided guidance and resolved disagreements. All authors approved it for publication.
